# A peripheral carboxysome component regulates cyanobacterial glycogen deposition

**DOI:** 10.1073/pnas.2607702123

**Published:** 2026-08-17

**Authors:** Joshua S. MacCready, Duncan M. Boren, María Santos-Merino, Luke A. Sharpe, Sigal Lechno-Yossef, Josh V. Vermaas, Daniel C. Ducat

**Affiliations:** ^a^ Michigan State University - Department of Energy, Plant Research Laboratory, Michigan State University, East Lansing, MI 48824; ^b^ Department of Biochemistry and Molecular Biology, Michigan State University, East Lansing, MI 48824

**Keywords:** carboxysome, glycogen metabolism, cyanobacteria, carbon metabolism, pentapeptide repeat protein

## Abstract

In cyanobacteria, carbohydrate products of the Calvin cycle can be utilized as precursors for cellular biosynthesis or stored as glucose polymers in glycogen granules. Cyanobacterial carbon fixation occurs in the carboxysome, while glycogen is synthesized in the cytosol via GlgC to support respiration and survival in the dark. Here, we identify a carboxysome-associated protein, Carboxysome Interacting Protein G (CipG), that appears to physically link these processes. CipG coassociates in cyanobacterial genomes with peripheral carboxysome structural proteins and localizes to carboxysomes in vivo via a process that requires conserved tryptophan residues. CipG coprecipitates with GlgC and is required for the recruitment of a subpopulation of GlgC to the surface of the carboxysome. CipG mutants display a glycogen deficiency, while CipG overexpression increases glycogen accumulation ~sixfold compared to wild type. Taken together, our results highlight a role for CipG in cyanobacterial energy metabolism and suggest an additional capacity for the carboxysome to scaffold key enzymes related to carbon partitioning in cyanobacteria.

## Significance

Carboxysomes are among the best-studied bacterial microcompartments with well-defined roles in specifying a subcellular microenvironment to promote the carbon-fixation reactions of photosynthesis. Here, we provide evidence that a previously uncharacterized protein, (CipG), associates with the β-carboxysome. We demonstrate that CipG has strong affinity for GlgC, an enzyme that controls a critical metabolic step for partitioning fixed carbon toward storage as glycogen. Our results further show that this carboxysome-associated protein has a regulatory function in the steady-state accumulation of carbon storage, perhaps by positioning a pool of GlgC to the perimeter of the carboxysome. Our results have broader implications that the carboxysome may have underappreciated roles beyond carbon fixation in the control of carbon partitioning downstream of the Calvin cycle.

**Fig. 1.**
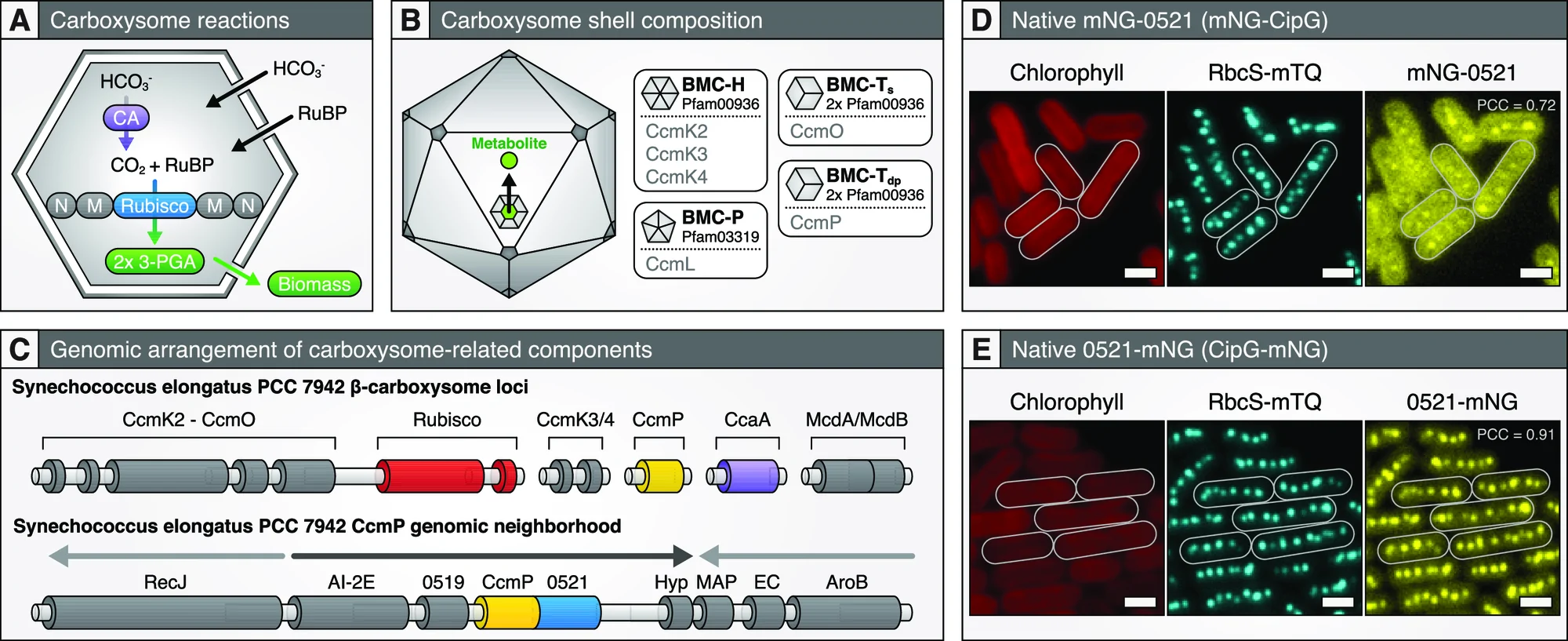
CipG is a carboxysome associated component. (*A*) Cartoon illustration of internal carboxysome reactions. (*B*) The carboxysome shell is composed of hexameric (BMC-H), pentameric (BMC-P), single trimeric (BMC-T_s_), and double-stacked permuted trimeric (BMC-T_dp_) proteins, each containing central pores that are proposed to mediate selective metabolite exchange between the carboxysome lumen and the cytosol. (*C*) Genomic arrangement of carboxysome components in *S. elongatus*. (*D*) Fluorescent tagging of CipG revealed that the native mNG-CipG fusion exhibits weak association with carboxysomes whereas, (*E*) the native CipG-mNG fusion demonstrates robust carboxysome localization. (Scale bar, 2 µM.)

Cyanobacteria are an evolutionarily and ecologically significant phylum of bacteria that perform oxygenic photosynthesis and contain specialized inorganic carbon-fixing bacterial microcompartments (BMCs) known as carboxysomes (1). Carboxysomes are a proteinaceous subcellular structure used to create a specialized microenvironment for Ribulose-1,5-bisphosphate carboxylase/oxygenase (Rubisco), an enzyme that is notorious for its slow catalytic rate and confused substrate utilization (2). Rubisco is concentrated within β-carboxysomes in part due to direct interactions with the luminal protein, CcmM (ccm = carbon concentrating mechanism). In turn, CcmM interacts with CcmN, a protein containing an encapsulation peptide domain that serves to nucleate and organize the shell proteins that form a semipermeable external barrier (3). By further encapsulating carbonic anhydrase, the carboxysome lumen can be enriched for Rubisco’s substrate CO_2_, favoring the carbon fixing conversion of ribulose-1,5 bisphosphate (RuBP) into two molecules of 3- phosphoglycerate (3-PGA) and minimizing the competing process of photorespiration (Fig. 1 *A* and *B*) (4).

Among BMCs, carboxysomes are relatively well-characterized in their structure and function, yet substantive questions directly related to the core function of this microcompartment remain. For example, carboxysome shell proteins are proposed to mediate metabolite trafficking between the lumen and the cytosol via pores situated at the centers of shell protein hexamers (BMC-H) and trimers (BMC-T) (Fig. 1*B*). In addition to the genes encoding the primary BMC-H (e.g., CcmK2) and BMC-T (CcmO) proteins, most cyanobacteria contain additional shell protein homologs hypothesized to facilitate the transport of a broader array of carboxysome substrates, products, and cofactors (e.g., RuBP, 3-PGA, 2-PG, ATP) (5). Yet, the permeability of shell proteins to primary substrates required for carboxysome function (e.g., HCO_3_^−^, CO_2_) are unknown, and computational models challenge the widely adopted hypothesis that carboxysome shells provide a selectively permeable barrier to HCO_3_^−^ and CO_2_ (6, 7). Furthermore, recent reports continue to expand the number of carboxysome-associated proteins beyond those that compose the structure of the compartment, including proteins involved in carboxysome positioning (8) and chaperone functions (9).

Herein, we describe a carboxysome-associated protein which associates with mature carboxysomes via a conserved, N-terminal domain. We find this protein is well conserved across many β-cyanobacteria and is involved in recruiting a rate-limiting enzyme involved in glycogen biosynthesis. Glycogen accumulation is misregulated in vivo within strains lacking native function of this carboxysome-associated protein. Taken together, our results suggest an intriguing hypothesis that cyanobacteria may exploit the carboxysome surface as a structural scaffold to regulate the partitioning of carbon derived from the Calvin Benson Bassham (CBB) cycle.

## Results

### A Gene Encoded Near CcmP Is Conserved in β-Cyanobacteria.

We conducted genomic neighborhood analyses to identify genes that consistently colocalize with carboxysome components across cyanobacterial genomes. In the model cyanobacterium *Synechococcus elongatus* PCC 7942 (*S. elongatus*), the majority of carboxysome components are encoded within the *ccm* operon, while a few accessory proteins are encoded at peripheral loci (Fig. 1*C*). Our analysis identified a gene that codifies for a highly conserved hypothetical protein (Synpcc7942_0521) across diverse cyanobacterial species, which is located upstream of the encoding region of the carboxysome accessory BMC-T shell protein, CcmP (Fig. 1*C*). Although the precise function of CcmP remains unresolved, it has been structurally determined to assemble into a “stacked trimer” that possesses a relatively large central pore chamber that may operate via an open/closed gating mechanism; a feature hypothesized to facilitate the selective passage of the larger metabolites such as the Rubisco substrate ribulose-1,5-bisphosphate (RuBP), and the carbon fixation product, 3-phosphoglycerate (3-PGA) (10).

We constructed N- and C-terminal mNeonGreen (mNG) fluorescent fusions of Synpcc7942_0521 to investigate its localization, utilizing a background with carboxysomes labeled by a Rubisco small subunit fluorescent fusion to mTurquiose2 (8) (RbcS-mTQ). In both strains, Synpcc7942_0521 colocalized with the carboxysome reporter (CR), although the C-terminal fusion exhibited significantly stronger colocalization (PCC = 0.91) compared to the N-terminal fusion (PCC = 0.72; Fig. 1 *D* and *E*). We designated this carboxysome component as CipG (Carboxysome Interacting Protein G). To our knowledge, the only prior evidence implicating a role for CipG was reported from a genome-wide screen designed to identify genes responsive to circadian rhythms, for which Synpcc7942_0521 was found to have moderate effect on cellular fitness under light–dark cycles (11). By contrast, the gene coding region immediately upstream of CcmP (Synpcc7942_0519) did not display colocalization with carboxysome puncta when this protein was visualized by N- or C-terminal fluorescent fusions (*SI Appendix*, Fig. S1 *A* and *B*).

### CipG Carboxysome Localization Is Mediated by Conserved Tryptophan Residues.

**Fig. 2.**
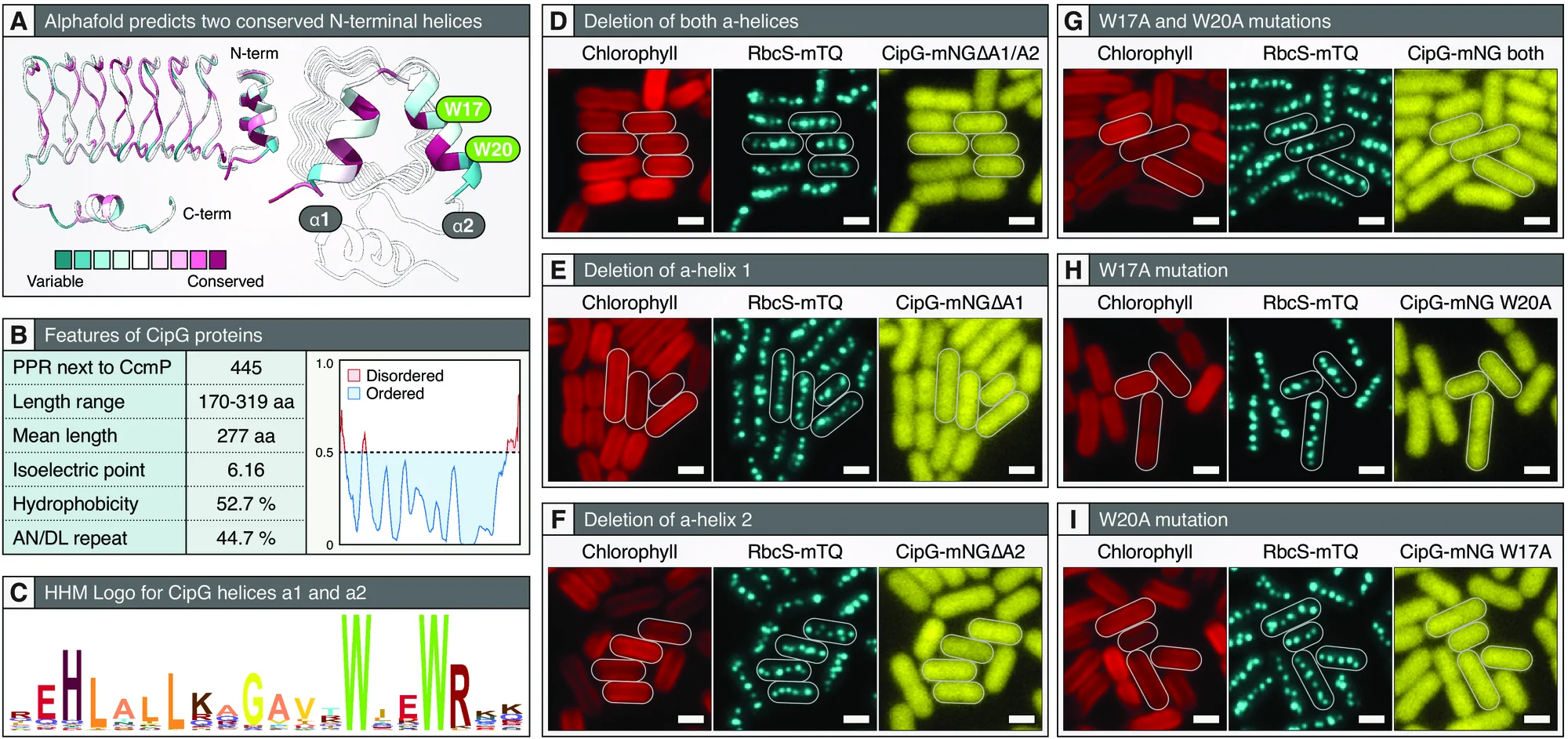
CipG carboxysome interaction is mediated by two highly conserved N-terminal α-helices. (*A*) AlphaFold predicts that CipG adopts a right-handed quadrilateral β-helical fold with two N-terminal α-helices containing two invariant tryptophan residues. Structural coloring reflects sequence conservation based on a multiple sequence alignment of 445 CipG homologs. (*B*) Features found among 445 CipG proteins encoded next to *ccmP* in their respective genome. (*C*) Hidden Markov Model (HMM) sequence logo representing the conserved residues of N-terminal α1 and α2 helices of CipG. (*D*) CipG-mNG fails to localize to carboxysomes in the absence of both α1 and α2 helices, as well as when either (*E*) α1-helix, or (*F*) α2-helix are individually removed. (*G*) CipG-mNG fails to localize to carboxysomes when both tryptophan residues are mutated (W17A and W20A), as well as when either (*H*) W17A, or (*I*) W20A mutations are introduced. (Scale bar, 2 µM.)

To gain further insight into CipG function, we conducted a comparative bioinformatic analysis of CipG homologs across sequenced cyanobacterial genomes. Using the Conserved Domain Database and AlphaFold structural prediction, we identified CipG as a member of the broad pentapeptide repeat protein (PRP) family (PF00805), characterized by at least eight consecutive pentapeptide repeats that fold into a right-handed quadrilateral β-helix, with each coil formed by four repeats (Fig. 2*A* and Movie S1) (12). Interestingly, data from the InterPro database revealed that although cyanobacteria represent only a small fraction of the bacterial entries (2.7%), they account for a disproportionately high percentage (20.7%) of PRPs, with many species possessing several homologs (*SI Appendix*, Fig. S2 *A* and *B* and Table S1). For example, in the genome of *S. elongatus*, we identified eight additional genes encoding PRPs. However, SignalP predictions suggest that seven of these are likely membrane-localized and the remaining PRP (Synpcc7942_0415) contains a relatively large nonrepeat domain, features that distinguish these proteins from CipG (*SI Appendix*, Fig. S1*C*).

Due to the abundance of cyanobacterial PRPs and their inherent repetitive sequence features, we restricted further analysis of potential CipG orthologs to only those genes colocalized with *ccmP*, resulting in the identification of 445 CipG homologs adjacent to *ccmP* (*SI Appendix*, Table S1). This analysis revealed significant variability in CipG length (170 to 319 AA), with a notable prevalence (44.7%) of Alanine-Asparagine-Leucine and Alanine-Aspartate-Leucine repeat motifs (Fig. 2*B*). The variation in CipG homolog protein length was reflected in a variable number of coils, ranging from 7 to 14 (*SI Appendix*, Fig. S3). Yet, all proteins contained two N-terminal α-helices, each harboring two invariant tryptophan residues within the motif WXXW(K/R) (Fig. 2*C* and *SI Appendix*, Fig. S3). Additionally, highly conserved histidine (H6 in *S. elongatus*) and arginine (R21) residues are predicted to participate in hydrogen bonding between the N-terminal α-helical domains and the right-handed β-helix pentapeptide repeat domain (*SI Appendix*, Fig. S4 and Movie S2).

The invariant tryptophan residues were intriguing in light of our previous finding that another protein associated with the carboxysome exterior, McdB, was recently determined to require an invariant tryptophan residue for carboxysome localization (13, 14). We therefore tested the localization of a number of mutant variants of CipG by altering the coding sequence in the context of its endogenous loci. C-terminally tagged fluorescent reporters of CipG mutants lacking either of the N-terminal α-helical regions (α1 and/or α2) showed a diffuse cytosolic localization pattern (Fig. 2 *D*–*F*) in contrast to the wildtype (WT) CipG localization (Fig. 1 *D* and *E*). Likewise, point mutation of the conserved tryptophan residues in *S. elongatus* (W17 and/or W20), prevented CipG-mNG from localizing to carboxysomes (Fig. 2 *G*–*I*).

### CipG Recruits a Subpool of GlgC to Carboxysomes.

**Fig. 3.**
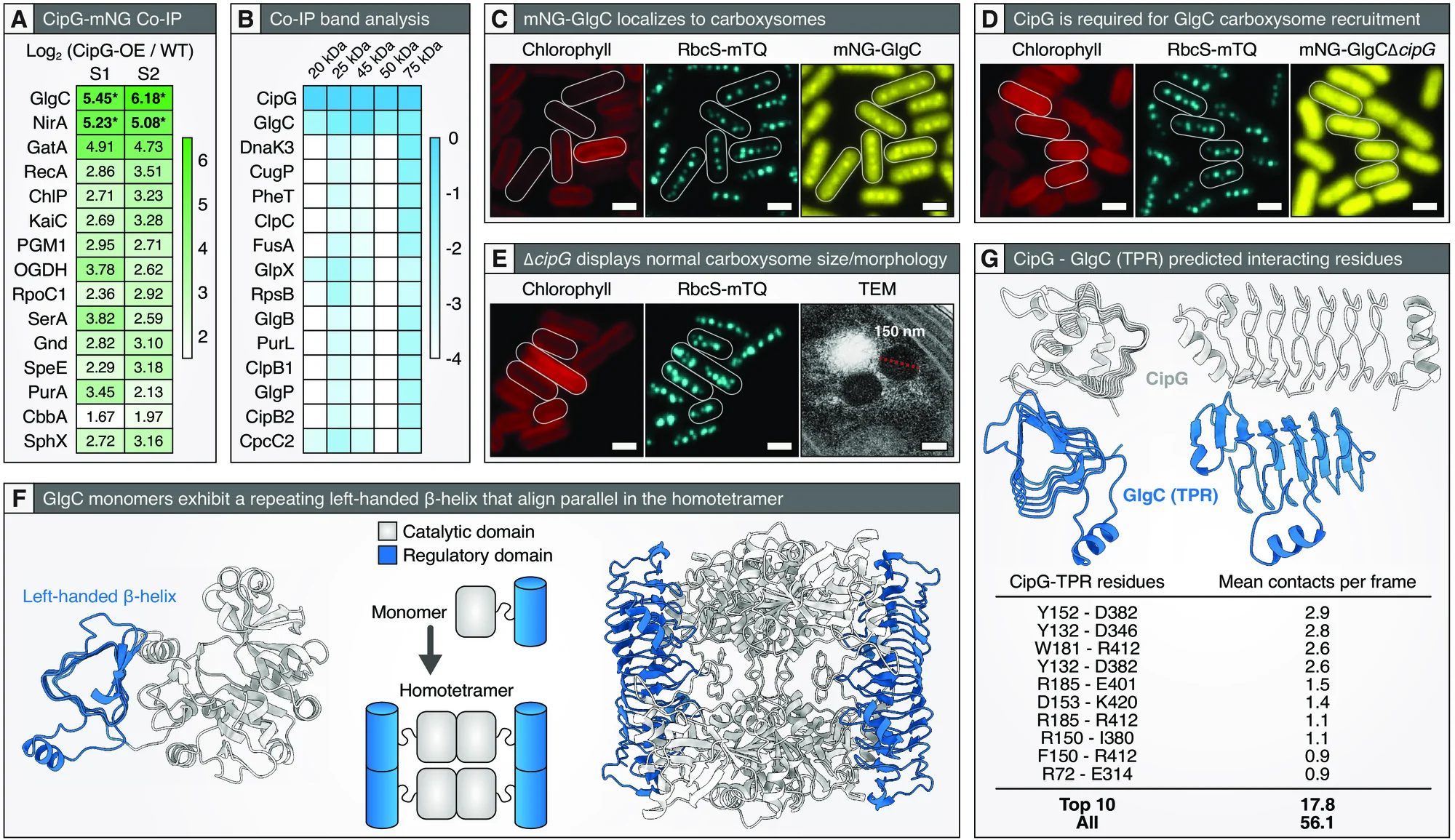
CipG recruits GlgC to carboxysomes. (*A*) Heatmap of enriched protein coprecipitating with CipG–mNG relative to WT samples. Proteins are ordered by statistical significance (Benjamini–Hochberg FDR–corrected q value) from a moderated statistical test. Asterisks (*) indicate proteins that remain significant after correction for multiple hypothesis testing. (*B*) Heatmap shows CipG-normalized log_2_ abundance values for the top-ranked proteins identified from excised gel bands of the indicated molecular weight, where each bin represents the mean of the log_2_-transformed CipG-normalized Quantitative Value (Normalized Total Spectra) averaged between the corresponding mNeonGreen–CipG and CipG–mNeonGreen fractions. Values are plotted on a negative scale, with lighter shading indicating lower abundance relative to CipG. (*C*) A subfraction of GlgC localizes to carboxysomes as identified by N-terminal labeling of the native copy of GlgC with mNG (yellow). A fluorescently tagged copy of the small subunit of rubisco (RbcS-mTQ) serves as a CR (blue). (*D*) GlgC localization to the carboxysome is absent in a background strain deleted for *cipG*. (*E*) Δ*cipG* strains exhibit normal carboxysome positioning within the resolution limits of light microscopy (*Left*) and TEM (*Right*). (*F*) Tetrameric structure of GlgC, highlighting the left-handed, repeat β-sheet helical domain. (*G*) Key residues predicted to mediate a CipG–GlgC interaction as determined by molecular dynamics simulations. (Scale bar, 2 µM.)

Pentapeptide repeat domains have not been previously reported to harbor inherent enzymatic function, but some distant family members have been implicated as having scaffolding functions for interacting proteins (12, 15). To identify potential interaction partners of CipG, we conducted a coimmunoprecipitation (Co-IP) assay by overexpressing CipG-mNG in *S. elongatus* and recovering the protein from cell lysates using α-mNG agarose beads. Initial proteomic analysis of the eluted factors identified multiple factors enriched in the CipG-mNG overexpression condition relative to WT controls, as ranked by limma-derived moderated t-statistics. Statistical significance was assessed using Benjamini–Hochberg–adjusted q-values, under which only nitrate reductase (NirA) and glucose-1-phosphate adenylyltransferase (GlgC) remained significant (Fig. 3*A* and *SI Appendix*, Fig. S5*A* and Table S2). Further insight into the most promising of these potential interacting proteins was gathered via SDS-PAGE analysis of coprecipitated proteins, revealing five primary bands, which were excised and submitted for further proteomic analysis (*SI Appendix*, Fig. S5*B*). Mass spec analysis of the two most prominent Co-IP protein bands (at ~45 to 50 kDa) revealed GlgC and CipG-mNG, respectively (Fig. 3*B*).

GlgC (also known as ADP-glucose pyrophosphorylase) is an enzyme that catalyzes the initial step of glycogen biosynthesis in cyanobacteria (16), but has no prior known association with the carboxysome. We visualized an N-terminal tagged fluorescent reporter of GlgC (mNG-GlgC) and determined that a subfraction is localized to carboxysomes (Fig. 3*C*). Furthermore, this localization is dependent on CipG, as the mNG-GlgC reporter was diffuse throughout the cytosol in a Δ*cipG* background (Fig. 3*D*). Furthermore, carboxysome morphology in a Δ*cipG* background appears normal when visualized by transmission electron microscopy (TEM) (Fig. 3*E*), suggesting that GlgC is not delocalized due to generalized loss of carboxysome integrity. Carboxysome carbon concentrating functions also appear intact in Δ*cipG* strains, which displayed no obvious growth deficiency relative to WT when grown under constant illumination and in ambient-CO_2_ conditions (*SI Appendix*, Fig. S6 *A* and *B*).

To gain further insight into the nature of the interaction between CipG and GlgC, we examined their structural features in greater detail. The structure of GlgC contains two distinct domains: an N-terminal glycosyltransferase-A–like catalytic domain and a C-terminal left-handed parallel β-helix, which modulates enzymatic activity through the binding of allosteric effectors such as fructose-1,6-bisphosphate, pyruvate, inorganic phosphate, or 3-PGA (17, 18) (Fig. 3*F*). In its native homotetrameric conformation, GlgC positions the β-helical domains on opposing faces of the catalytic core (Movie S3). These helical domains align between two subunits in the active, tetrameric form of GlgC in vivo in cyanobacteria (Fig. 3*F*) (19, 20). This architecture features a five–amino acid repeat that, despite substantial divergence in primary sequence across cyanobacterial species, adopts a conserved structural arrangement that bears a striking resemblance to the four-sided helix characteristic of pentapeptide repeat domains (*SI Appendix*, Fig. S7 and Movies S4 and S5).

### Evaluation of Putative CipG–GlgC Interface via Molecular Dynamics.

Preliminary analysis via Alphafold3 frequently predicted potential protein–protein interfaces including contacts between CipG and the helical domain of GlgC. We therefore turned to molecular dynamics simulations to probe the possibility that these structurally similar domains could mediate GlgC–CipG interactions. To reduce the computational load for atomistic simulations, we isolated GlgC’s left-handed β-sheet helical domain (hereafter TPR) and evaluated its potential interactions with CipG. Molecular dynamics simulations were run from a bound starting pose of the CipG–TPR dimeric complex. Examining the RMSD of the dimeric complex in reference to simulation starting poses, there are fluctuations of up to 8 Å (*SI Appendix*, Fig. S8*A*), without dissociation of the interface. This RMSD is larger than the monomeric CipG and TPR proteins alone (*SI Appendix*, Fig. S8 *B*–*D*) and can be ascribed to reorientation between the two individual monomers, largely representing a sliding of the two proteins with respect to one another. Across 1 µs simulations, the CipG–TPR dimer maintains a high number of contacts, even during the largest fluctuations observed (*SI Appendix*, Fig. S8*A*).

Several residues in CipG and TPR are overrepresented in contributing protein–protein contacts across simulation datasets of the CipG–TPR interaction (Fig. 3*G* and *SI Appendix*, Fig. S9). CipG aromatic residues Y132, Y152, W181, and F150 are involved in 40% of protein–protein contacts while GlgC residues R412, D382, S413, and M346 are the most represented contacts over the course of the simulation (Fig. 3*G* and *SI Appendix*, Fig. S9). This represents an interaction between face 1 of the columnar pentapeptide repeat domain as well as contributions from C-terminal residues of CipG to the interaction interface. The highest-RMSD binding poses of the simulations typically feature rotations of the GlgC TPR domain that separate the N terminus away from CipG, without breaking C-terminal contacts. Because the CipG and GlgC TPR domain do not spontaneously dissociate over computationally tractable timescales, Replica Exchange Umbrella Sampling (REUS) simulations were used to probe the transition states along the protein dissociation reaction and estimate protein–protein affinity. The free energy profile along this coordinate (*SI Appendix*, Fig. S10) indicates that protein–protein association is strongly favored. The bound state, where the protein centers of mass are only separated by 25 Å, is 12.8 kcal/mol lower in energy than the unbound state. This free energy change, together with an R value of 1.986 kcal/(mol*k) and a system temperature of 303 K, yields a K_d_ of 672 picomolar for the CipG–TPR dimer. Based upon this predicted affinity, the bound state of CipG would be highly likely within the cell, as even a single copy of each protein per cell would represent a concentration higher than this binding affinity.

### CipG Regulates Glycogen Accumulation in *S*. *elongatus*.

**Fig. 4.**
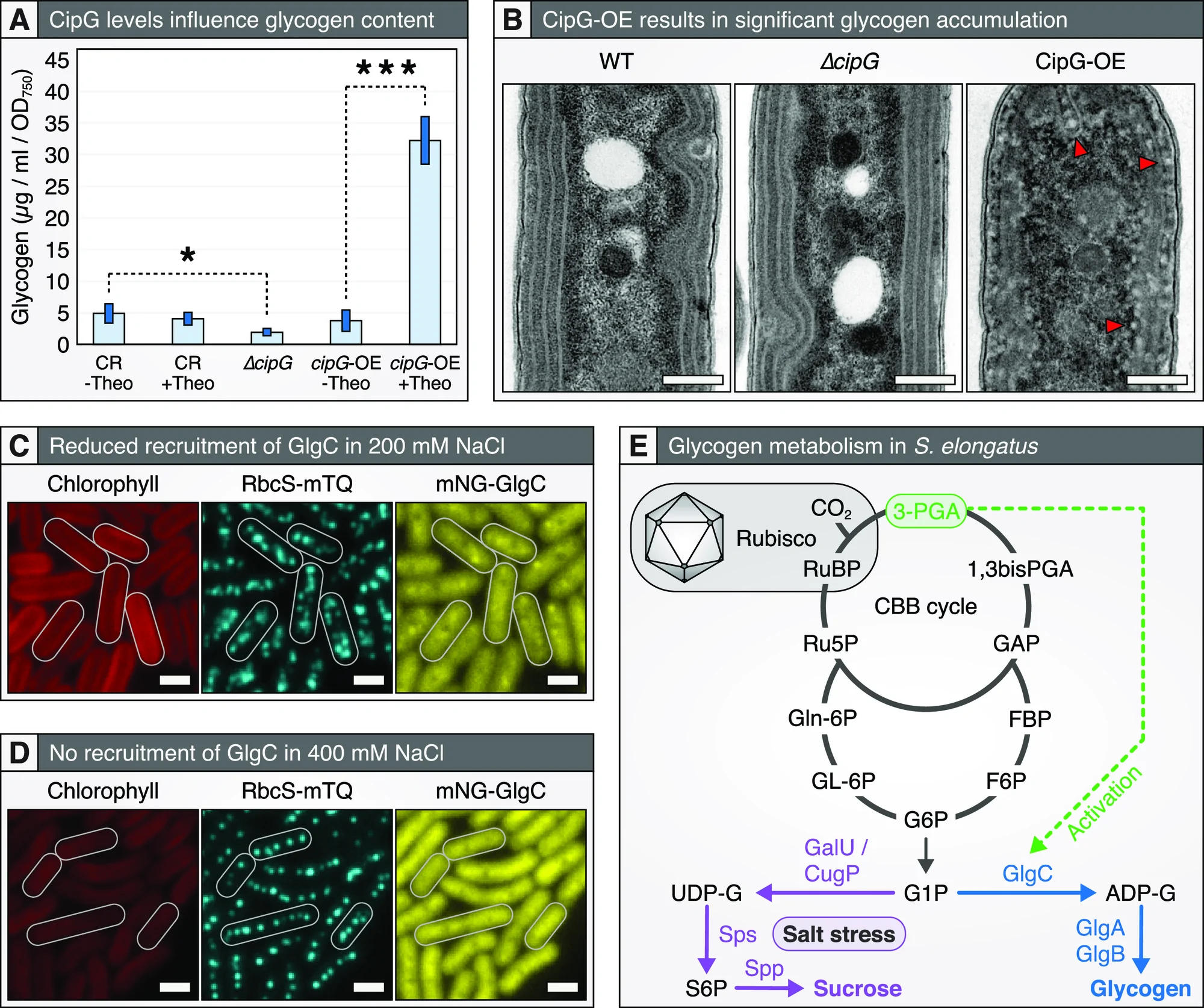
CipG influences GlgC activity in vivo. (*A*) The steady state level of accumulated glycogen was measured from *S. elongatus* strains grown under constant light. Glycogen content of CR only control strains with and without theophylline (Theo) were compared to strains deleted for *cipG* (Δ*cipG*) and for strains with an extra copy of *cipG* driven by an inducible promoter responsive to Theo. Averages of three independent biological replicates are shown with ± SD reported with error bars. Significance was calculated by unpaired Student’s *t* test. *P < 0.05, ***P < 0.001. (*B*) Representative TEM images of *S. elongatus* strains as in *A*. (Scale bar, 250 nM.) Localization of a natively tagged GlgC reporter (mNG-GlgC) background when cells were cultivated with 200 mM (*C*) or 400 mM (*D*) of additional NaCl to induce salt stress. A fluorescently tagged copy of the small subunit of rubisco (RbcS-mTQ) serves as a CR (blue), while chlorophyll a autofluorescence is displayed in red. (*E*) Schematic representation of glycogen and sucrose synthesis pathways originating from glucose-1-phosphate. (Scale bar, 2 µM.)

We next evaluated the possibility that GlgC’s activity is influenced by its association with CipG and/or association with carboxysomes. To control *cipG* expression levels we genomically integrated an extra copy of *cipG* under the control of a tunable theophylline riboswitch (21) (hereafter *cipG*-OE). We measured steady state glycogen content under constant light growth conditions for WT, Δ*cipG*, and *cipG*-OE strains. We observed a ~50% reduction in glycogen levels in the *∆cipG* mutant compared to WT (Fig. 4*A*). By contrast *cipG*-OE lines exhibited a >sixfold increase in glycogen accumulation upon the addition of theophylline inducer (Fig. 4*A*). Glycogen hyperaccumulation was further confirmed via TEM imaging, which revealed a substantial increase in electron-permeable puncta associated with glycogen granules; in addition to cytosolic glycogen, granules were frequently positioned near and within thylakoid membranes, whereas these were rarely observed in ∆*cipG* and WT backgrounds (Fig. 4*B*). Strikingly, while glycogen levels were depleted in *∆cipG* mutants under routine growth conditions, this relationship was reversed under salt stress, where Δ*cipG* cells accumulated more glycogen than WT at both 200 mM and 400 mM NaCl (*SI Appendix*, Fig. S11*A*). Furthermore, glycogen accumulation was unchanged between WT and Δ*cipG* lines under conditions of nitrogen depletion (*SI Appendix*, Fig. S11*A*), an environmental perturbation that has been well established to result in dramatically increased glycogen deposition via alternative metabolic regulation.

To determine if the recruitment of GlgC to the carboxysome is a regulated process that may impact the observed changes in glycogen deposition, we evaluated GlgC localization under different growth conditions. We focused on salt stress because this environmental change is known to redirect carbon flux away from glycogen synthesis toward sucrose production. We examined GlgC localization under osmotic stress and observed a progressive, salt-dependent dissociation of natively tagged mNG-GlgC from carboxysome puncta as NaCl concentrations increased from 200 mM to 400 mM (Fig. 4 *C* and *D*). These results indicate that GlgC recruitment to the carboxysome periphery is dynamically regulated in response to environmental conditions, with potential implications for the regulation of carbon partitioning in cyanobacteria. Together with the reduced glycogen accumulation observed under standard growth conditions, these findings suggest that CipG may function in the regulation of glycogen homeostasis, rather than serving solely as a positive regulator of glycogen accumulation.

## Discussion

Herein, we present evidence for a carboxysome-associated protein that is conserved across most β-cyanobacterial species, and which recruits an enzyme related to central carbon metabolism to the carboxysome periphery. CipG (carboxysome interacting protein G) is a member of the poorly characterized PRP family widespread in many prokaryotic species and especially prevalent in cyanobacteria, algae, and plants [PF00805; (12)]. CipG has an N-terminal domain with two α-helices, which contains two invariant tryptophan residues essential for its localization to the carboxysome (Fig. 2). In *S. elongatus*, we find CipG recruits a subfraction of GlgC to the carboxysome periphery. Our data further suggest that CipG may influence glycogen accumulation through GlgC, thereby implying that, beyond their well-established role in the cyanobacterial ccm, carboxysomes may also participate in the regulation of partitioning for fixed carbon.

Multiple lines of evidence suggest that CipG and GlgC interact in vivo, and that this association promotes glycogen accumulation. In pulldown experiments, GlgC coelutes at a nearly equimolar ratio with CipG in coprecipitation studies (Fig. 3 *A* and *B* and *SI Appendix*, Fig. S5). A natively tagged GlgC reporter colocalizes with carboxysome puncta in a manner requiring *cipG* (Fig. 3 *C* and *D*). Furthermore, *cipG* overexpression significantly enhances glycogen accumulation while deletion of *cipG* suppresses glycogen accumulation under routine cultivation conditions (Fig. 4 *A* and *B*). Molecular dynamics simulations predict a strong interaction between GlgC and CipG mediated directly by key residues on face 1 of CipG’s pentapeptide repeat domain (Fig. 3*G* and *SI Appendix*, Figs. S8–S10). We note that this predicted interaction interface would position CipG near established regulatory residues involved in allosteric regulation of GlgC (17, 22, 23) (*SI Appendix*, Figs. S7, S8, and S11*B*). However, this predicted interface would also likely require a significant rotation of GlgC’s TPR domain to avoid steric clash, therefore additional experimental validation via in vitro binding assays or mutational studies would bolster this proposed interaction.

Our data cannot discriminate between the possibilities that CipG regulates GlgC function directly or by influencing its cellular localization. An intriguing hypothesis is that positioning GlgC could alter its microenvironment in ways that impact enzymatic activity and glycogen biosynthesis. In this context, 3-PGA is a well-established allosteric activator of ADP-glucose pyrophosphorylase (GlgC) in both plants (24, 25) and cyanobacteria (26, 27), unlike homologous enzymes in other bacteria. Indeed, recent in vitro characterization has reported that cyanobacterial GlgC activity is enhanced up to eightfold by 3-PGA (19). 3-PGA is the primary product of the Rubisco housed within the carboxysome lumen, raising the possibility that the lumen and vicinity of enzymatically active carboxysomes may have elevated concentrations of 3-PGA relative to the bulk cytosol. Furthermore, the stacked, pseudohexameric structure of CcmP creates a gated channel with favorable physiochemical properties to facilitate transport of CBB intermediates RuBP and 3-PGA (10), although the pore of other carboxysome shell proteins may also permit larger metabolite transport provided sufficient conformational flexibility (6, 28). Given that *cipG* is consistently encoded in the genomic neighborhood of *ccmP* (Figs. 1*B* and 2*C* and *SI Appendix*, Fig. S3), it is tempting to speculate these two carboxysome genes may share related functions regarding processing of 3-PGA synthesized in the carboxysome.

The delocalization of GlgC under salt stress (Fig. 4 *C* and *D*) suggests its attachment to the carboxysome may be regulated under certain environmental conditions. In many species of cyanobacteria, including *S. elongatus*, salt stress leads to a redirection of carbon resources toward the hyperaccumulation of sucrose as a protective compatible solute (29, 30). Sucrose synthesis proceeds by the conversion of glucose-1 phosphate (G1P) to UDP-glucose, catalyzed by UDP-glucose pyrophosphorylases such as GalU or the non-GalU enzyme CugP, and subsequent activities of sucrose phosphate synthase (SPS) and sucrose-phosphate phosphatase (SPP). Because of their common utilization of the cellular pool of G1P in the commitment steps, synthesis of glycogen and sucrose can be conceptualized as competing for central carbon metabolic precursors (Fig. 4*E*). Indeed, we observe that Δ*cipG* mutants grown under moderate salt stress do not exhibit the characteristic decline in glycogen content reported in WT as carbon resources are reallocated away from glycogen and toward sucrose (*SI Appendix*, Fig. S11*A*). Intriguingly, a CugP-like protein (Synpcc7942_0498) was the fourth most abundant protein identified in our Co-IP band analysis (Fig. 3*B*), and AlphaFold-based structural prediction revealed that unlike GalU, CugP-like proteins adopt a left-handed parallel β-helix reminiscent of the regulatory domains of GlgC (*SI Appendix*, Fig. S11*C*). These observations raise the possibility that CipG may also interact with CugP-like proteins, thereby modulating the partitioning of G1P between CugP and GlgC, or, more broadly, that additional PRPs may interact with accessory proteins containing triangular β-helical domains.

The PRP family of proteins remain relatively poorly understood despite their abundance in the prokaryotic kingdom (12). Since their discovery (31), PRP homologs have been implicated in a wide range of regulatory processes, including: i) heterocyst glycolipid localization (HglK), ii) manganese uptake (RfrA), iii) gyrase inhibition (MfpA, AlbG, and Qnr), and iv) Ubiquitin E3 Ligation (SopA and NleL) (32–35). PRPs are broadly classified into three groups, where groups 1 and 2 (36) have been the most intensively studied and are proposed to interact with DNA gyrase and topoisomerase through a mechanism whereby the pentapeptide helical repeat resembles key structural features of DNA (34). By contrast, PRPs from group 3 are particularly diverse in their functions and are overrepresented in cyanobacterial genomes (12). This work describes a function for group 3 PRPs in relation to carboxysomes and central carbon metabolism, yet the apparent scaffolding functions of CipG may be shared with other PRP homologs involved in distant processes. Namely, a common feature of PRPs is that the β-sheet fold outwardly projects the side chains of three amino acids (at positions i^−1^, i^+1^, and i^+2^ of each repeat), such that these residues are involved in mediating protein–protein contacts across a diversity of protein partners (12). Indeed, the modular nature of the pentapeptide repeat has been proposed to be particularly adaptable for evolving and engineering of cellular scaffolding functions (12, 37).

More broadly, CipG features may highlight a strategy to localize enzymes to the carboxysome surface. The N terminus of CipG contains an α-helical domain containing two invariant tryptophan residues apparently critical to mediate carboxysome association (Fig. 2). Although the mechanism remains unknown, this feature is similar to an absolutely conserved tryptophan residue in the protein McdB (maintenance of carboxysome distribution B) which is also required for McdB to attach the outer face of the carboxysome (14, 38). An intriguing possibility for future research is that CipG and related PRPs could be used (in natural or engineered systems) to recruit other enzymes to the surface of carboxysomes to utilize the ordered BMC shell surface as a scaffold to organize and regulate other enzymes. Proteins that naturally bear left-handed parallel β-helix domains similar to that of GlgC TPR, such as the CugP enzyme involved in sucrose biosynthesis (Fig. 4*E* and *SI Appendix*, Fig. S11*C*), may be especially intriguing to investigate in this regard.

## Materials and Methods

### Phylogenetic Inference.

CipG sequence alignment was performed using MAFFT 1.3.7 under the E-INS-I algorithm and BLOSUM62 scoring matrix. A phylogenetic tree was then inferred with IQTree 2.3.6 (1000 SH-aLRT bootstrap replicates) using Model Finder Plus (MFP) to determine the optimal evolutionary model

### Construct Design.

All constructs in this study were synthesized using Gibson Assembly (39) with double-stranded DNA. DNA sequences were ordered commercially to be synthesized (Integrated DNA Technologies), cloned into the target vector, and confirmed by sequencing. Homologous recombination was used to insert genetic material into the genome of *S. elongatus* by standard methodology (40), utilizing ~500 to 1,000 bp of flanking DNA upstream and downstream of the targeted insertion site. Genetic constructs were typically inserted to modify the native locus or at neutral site 2, to facilitate homologous recombination into the desired location (40). CipG overexpression was regulated through the use of a synthetic riboswitch driven by the P*_trc_* promoter and lacking *lacI* (21, 41). The CR, P*_rbcs_*::RbcS-mTQ, was inserted at neutral site 1 as previously described (38).

### Culture Conditions and Transformations.

All *S. elongatus* cultures were cultivated in 125 mL baffled flasks (Corning) containing 50 mL BG-11 medium (Sigma), buffered with 1 g/L HEPES to a pH of 8.3. Cultures were incubated in a Multitron II (Infors HT) shaker-incubator under the following conditions: 120 µmol m^−2^ s^−1^ light intensity, 130 RPM shaking, 32 °C, and 2% CO_2_. Plasmid cloning was carried out in *Escherichia coli* DH5α chemically competent cells (Invitrogen). All *S. elongatus* transformations were performed as previously described (40). Cells were plated on BG-11 agar supplemented with either 12.5 mg/mL kanamycin, 12.5 mg/mL chloramphenicol, or 25 mg/mL spectinomycin. Single colonies were selected and transferred into 96-well plates containing 300 μL of BG-11 medium supplemented with the corresponding antibiotic concentrations. Cultures from wells containing positive genetic insertions were subsequently transferred to 125 mL baffled flasks with 50 mL BG-11 medium and subjected to progressively higher antibiotic concentrations until complete segregation was confirmed.

### Induction and Glycogen Measurements.

For CipG overexpression, 1 mM of theophylline was added to 125 mL baffled flasks containing 50 mL of BG-11 medium and incubated for 24 h. For measurement of glycogen content under nitrogen depleted conditions cultures of the indicated genetic backgrounds were pelleted by centrifugation and washed 3× with BG11 medium lacking NaNO_3_ (BG11_0_). Cells were then resuspended in BG11_0_ and grown for 24 h under the previously indicated growth conditions prior to assessment of glycogen content. For salt stress treatment, cultures of the indicated genetic backgrounds were grown as previously indicated and an appropriate volume of 5 M NaCl was added to achieve a final concentration of 0, 200, or 400 mM NaCl at time = 0. Glycogen content was assessed for salt-treated cultures at 24 h.

Glycogen content was monitored from the indicated conditions as described previously (42). Briefly, cell pellets were resuspended in 200 µL of 30% (w/v) KOH and incubated at 95 °C for 2 h. 600 µL absolute ethanol was added to samples prior to incubation at −20 °C. Following precipitation, centrifuged pellets were desiccated, then resuspended in ddH2O for analysis via a commercially available Glycogen assay kit (EnzyChrom, San Jose, CA).

### Fluorescence Microscopy.

Live-cell microscopy was performed using exponentially growing cultures. A 2 mL aliquot of culture was centrifuged at 5,000 × g for 30 s, resuspended in 50 µL of BG-11, and 2 µL of the suspension was applied to a square pad composed of 2% agarose and BG-11 on a glass slide. Images were acquired using a Zeiss Axio Observer A1 microscope (100× objective, 1.46 NA) equipped with an Axiocam 503 mono camera. Image analysis was performed, and Pearson correlation coefficients (PCC) were calculated, using Fiji v2.15.1.

### TEM.

Cyanobacterial cultures were serially backdiluted in BG-11 medium for three consecutive days and grown to a final optical density of OD_750_ = 0.7. CipG overexpression was induced by adding 1 mM theophylline to cultures following backdilution on the second day. Cells were pelleted by centrifugation at 5,000 × g for 3 min, then fixed overnight at 4 °C in a solution of 2.5% formaldehyde and 2.5% glutaraldehyde in 0.1 M sodium cacodylate buffer (pH 7.4). Samples were subsequently embedded in 2% high-purity agarose and sectioned into approximately 1 mm cubes. After three washes with 0.1 M sodium cacodylate buffer, cells were resuspended in a solution of 1% osmium tetroxide and 1.5% potassium ferrocyanide and incubated overnight at 4 °C. After incubation, cells were repeatedly washed with HPLC-quality H_2_O. Cells were then suspended in 1% uranyl acetate and microwaved for 2 min using a MS-9000 Laboratory Microwave Oven (Electron Microscopy Science), decanted, and washed until clear. Cells were dehydrated in increasing acetone series (microwave 2 min) and then embedded in Spurr’s resin (25% increments for 10 min each at 25 °C). A final overnight incubation at room temperature in Spurr’s resin was done, then cells were embedded in blocks which were polymerized by incubation at 60 °C for 3 d. Thin sections of approximately 50 nm were obtained using an MYX ultramicrotome (RMC Products), poststained with 1% uranyl acetate and Reynolds lead citrate and visualized on a JEOL 1400 Flash transmission electron microscope (JEOL) equipped with an Orius SC200-830 CCD camera (Gatan). Contrast correction was performed using Fiji v2.15.1.

### Culture Growth and Cell Lysate Preparation.

Triplicate cultures of *S. elongatus* WT and CipG-mNG overexpressing in NS2 from Ptrc-RS, were backdiluted to OD_750_ of 0.3 for 3 d. In the last day the CipG-mNG was induced by 1 mM IPTG and 1 mM theophylline. The cultures were grown for an additional 24 h. 50 mL cultures of each strain were harvested, suspended in 1 mL BG-11 and 0.5 mL of cell suspension was transferred to an Eppendorf tube. The cultures were harvested, and the remaining liquid was decanted. Cell pellets were frozen at −80 °C overnight. The cell pellets were then desiccated in a lyophilizer overnight. Lyophilized cells were broken by bead beating with 3 cycles or 30 Hz for 1 min on and one min off in a TissueLyser in the presence of 4 to 5 glass beads (2 mm diameter). Pulverized cells were suspended in 520 mL lysis buffer (50 mM Tris, pH 8, 1 mM DTT, 0.3 mM EDTA, 50 mM NaCl, 1% Triton X-100, and 1X Pierce EDTA-free protease inhibitor). Cell debris were removed by slow centrifugation at 5,000 rpm for 5 min, and lysates were clarified by an additional 5 min centrifugation at 14,000 rpm. Protein concentration in cell lysates was estimated by BCA Pierce assay (Thermo) according to manufacturer instructions.

### Co-IP—Total Protein.

mNeonGreen-trap magnetic agarose beads (Chromotek–ntma) were used according to manufacturer instructions. Briefly, a total of 25 µL bead slurry was washed in manufacturer recommended dilution buffer and 500 µL cell lysate containing a total of 500 µg protein were incubated with the beads overnight at 4 °C. Two biological replicates of the WT control and the CipG-mNG lysates were tested. The beads were washed three times and proteins were eluted using 80 µL of 2× SDS-sample buffer. The beads were boiled for 5 min and beads were removed. Samples were analyzed by SDS-PAGE and visualized by silver straining. Remaining sample was electrophoresed into the stacking gel of an SDS-PAGE, visualized by Coomassie staining and the destained bands were cut from a gel and submitted for mass spectroscopy analysis.

### Co-IP—Band Analysis.

Cultures of mNG-CipG and CipG-mNG were grown in 100 mL BG-11 medium in 250 mL baffled flasks and backdiluted to an OD_750_ of 0.3 for 3 d. On the final day, cultures were induced with 1 mM theophylline. After 24 h of induction, cells were harvested by centrifugation at 5,000 × g for 10 min and resuspended in 20 mL lysis buffer (Chromotek) supplemented with protease inhibitor. Cells were lysed by passing them three times through a continuous-flow cell disruptor at 35,000 PSI, followed by a final wash with 10 mL dilution buffer (Chromotek) containing protease inhibitor. An additional 20 mL of dilution buffer was added to bring the total volume to 50 mL. Lysates were centrifuged at 5,000 × g for 10 min, and the supernatant was transferred to a fresh 50 mL conical tube. Pre-equilibrated mNeonGreen-Trap agarose beads (100 µL; Chromotek–ntak) were added to the supernatant and incubated overnight on a rocker at 4 °C. Beads were then washed eight-times with wash buffer and centrifugation (Chromotek), and bound proteins were eluted with 40 µL of 2× SDS sample buffer. Samples were boiled for 5 min, and beads were removed. Samples were analyzed by SDS-PAGE and visualized by Coomassie and/or silver staining. Bands of interest were excised and submitted for mass spectrometry analysis.

### Proteolytic Treatment and LC/MS/MS Analysis of Co-IP Proteins.

Gel bands were digested in-gel according to Shevchenko et al. (43) with modifications. Briefly, gel bands were dehydrated using 100% acetonitrile and incubated with 10 mM dithiothreitol in 100 mM ammonium bicarbonate, pH ~ 8, at 56 C for 45 min, dehydrated again, and incubated in the dark with 50 mM chloroacetamide in 100 mM ammonium bicarbonate for 20 min. Gel bands were then washed with ammonium bicarbonate and dehydrated again. Sequencing grade modified trypsin was prepared to 0.005 μg/μL in 50 mM ammonium bicarbonate and ~100 μL of this was added to each gel band so that the gel was completely submerged. Bands were then incubated at 37C overnight. Peptides were extracted from the gel by water bath sonication in a solution of 60% Acetonitrile (ACN)/1% Trifluoroacetic acid (TFA) and vacuum dried to ~2 μL.

Dried peptides were resuspended in 2% ACN/0.1% Trifluoroacetic acid to 20 μL. From this, an injection of 2 μL was automatically made using a Thermo (www.thermo.com) EASYnLC 1000 onto a Thermo Acclaim PepMap RSLC 0.1 mm × 20 mm C18 trapping column and washed for ~5 min using Buffer A. Bound peptides were then eluted onto a Thermo Acclaim PepMap RSLC 0.075 mm × 150 mm C18 resolving column over 35 min with a gradient of 5% B to 38% B in 24 min. After the gradient the column was washed with 90%B for the duration of the run (Buffer A = 99.9% Water/0.1% Formic Acid, Buffer B = 80% Acetonitrile/0.1% Formic Acid/19.9% Water) at a constant flow rate of 400 nL/min.

Eluted peptides were sprayed into a ThermoScientific Q-Exactive mass spectrometer (www.thermo.com) using a FlexSpray spray ion source. Survey scans were taken in the Orbi trap (35,000 resolution, determined at m/z 200) and the top 10 ions in each survey scan were then subjected to automatic higher energy collision induced dissociation (HCD) with fragment spectra acquired at a resolution of 17,500.

### Proteomic Data Analysis.

Label free quantification (LFQ) was conducted in MaxQuant as described (44). LFQ intensity values were log2-transformed in Perseus and missing data were imputed using normal distribution. *t* test was performed in Perseus comparing Samples and Controls. Raw data presenting proteomic analysis are provided in *SI Appendix*, Table S2.

### Computational System Construction.

Our goal was to build a model for both proteins of interest interacting with one another. The initial pose for interacting proteins was generated using Alphafold3 (45), from uniprot sequences Q31QN4 (GlgC) and Q31QW6 (CipG).

The proposed binding region of GlgC is predicted to be shielded by the hydrophobic protein interior. To best expose the region for binding, we chose to simulate a truncated form of GlgC, with the first 296 residues removed. The system was solvated and ionized using the VMD tools SOLVATE and AUTOIONIZE. The final simulation system had dimensions of 12 nm by 12 nm by 12 nm. This size was chosen to optimize simulation speed while allowing realistic protein dynamics unaltered by periodic boundary effects.

### Molecular Dynamics Simulations.

Equilibrium molecular dynamics simulations were carried out using NAMD 3.0.1 (46, 47) using TIP3 water and CHARMM36 protein force fields (47, 48), with a 12 Å cutoff. Particle mesh Ewald was used with 1.2 Å grid spacing for long range electrostatics (49, 50). To allow 2 fs timesteps, the SETTLE algorithm was used to fix the length of covalent bonds to hydrogen atoms (51). The system was simulated in an NPT ensemble using a Langevin thermostat and an isotropic barostat to maintain 303 K and 1 atm, respectively. To sample movements within the dimerized state, five equilibrium simulation replicates (1,000 ns each) were run from a bound starting state.

Analysis for the equilibrium trajectories was done in VMD 1.9.4a58 (52). Principally, we used python and tcl scripts to access the built-in analysis routines to quantify hydrogen bonds (with distance and angle cutoffs of 3.2 Å and 30 degrees, respectively) and the rmsd from the starting configuration. We track contacts between GlgC and CipG using a distance weighted contact equation, originally developed by Sheinerman and Brooks (53).

C_i =∑_{j∈B}^ {}⧄{1}{1+exp ⊢(5”\AA^ {-1}⊢(d_{ij}-4”\AA⊣)⊣)}.

In this equation, *Ci* is the number of contacts being made by heavy atom *i* at a given timestep, *B* is the set of all heavy atoms in the system that are not *i*, and *dij* is the distance between heavy atom *i* and heavy atom *j*.

### REUS.

No unbinding was observed in the equilibrium simulations, therefore the strength of the binding between TPR and PRP was quantified via REUS to sample the unbinding path. Umbrella sampling was achieved by defining 64 centers evenly spaced between 25 and 55 Å of distance between proteins, measured from protein centers of mass. Each replica was run to 25 ns, for an aggregate of 1,600 ns of simulation time. To calculate dissociation probability, the relationship between free energy and dissociation constant was be expressed as

$$k_{d}=e^{\frac{\Delta G}{\mathrm{RT}}}.$$

By comparing the bound state, where the protein centers of mass are only separated by 25 Å, the unbound state’s free energy change was determined to be 12.8 kcal/mol lower in energy. Using the R value of 1.986 kcal/(mol*K) and a system temperature of 303 K, yields a Kd of 672 picomolar for the GlgC truncation and CipG complex.

## Supplementary Material

Appendix 01 (PDF)

Dataset S01 (XLSX)

Dataset S02 (XLSX)

Movie S1. 3D projection of Fig. 2A showing conservation of CipG residues.

Movie S2. 3D projection of Fig. S4B showing predicted hydrogen bonding residues of CipG N-terminal helices.

Movie S3. 3D projection of GlgC with regulatory β-helices highlighted in blue.

Movie S4. 3D projection of Fig. S7 showing residue conservation across 608 cyanobacterial GlgC homologs.

Movie S5. 3D projection of Fig. S7 highlighting residue conservation of β-helices across 608 cyanobacterial GlgC homologs.

## Data Availability

Study data are included in the article and/or supporting information.
