# Supplementary material for: A peripheral carboxysome component regulates cyanobacterial glycogen deposition
Source: Proc Natl Acad Sci U S A. 2026 Aug 17;123(34):e2607702123. doi: 10.1073/pnas.2607702123 (PMC13506006; doi:10.1073/pnas.2607702123)
Supplement: Supplementary file 1 [file pnas.2607702123.sapp.pdf]

PNAS Template for Supporting Information

This PNAS template for supporting information (SI) may be used to organize your SI Appendix. **Once formatted, this first page should be deleted.** The template is intended to provide a clearly organized PDF that readers can easily navigate to specific information, figures, and tables. Movie files or large datasets must be presented as separate files. Further information is available in the PNAS Author Center.

Using the Template
Paste the appropriate text (title, author list, and corresponding authors) into the cover page. You may maintain the template formatting or reapply the styles after pasting your text into the template. On the cover page, complete the relevant description of the SI and delete text that does not apply.

Supporting information for Brief Reports is limited to extended methods, essential supporting datasets, and videos (no additional tables or figures).

SI figures should be placed on separate pages with a figure legend set immediately below each figure. Table titles should be set immediately above each SI table. Note that tables extending beyond the width of the page can be included; nonstandard page sizes are accepted as part of your SI Appendix file.

All references cited in the SI Appendix should be included in a separate reference list at the end of this file.

Save your completed SI Appendix file as a PDF. SI will be published as provided by the authors; it will not be edited or composed.

Delete this first page prior to submission.

Updated August 2025

Supporting Information for:

A novel peripheral carboxysome component regulates cyanobacterial glycogen deposition

Joshua S MacCready[1,x], Duncan M. Boren[1,2], María Santos-Merino[1], Luke A. Sharpe[1,2], Sigal Lechno-Yossef[1], Josh V. Vermaas[1,2], and Daniel C Ducat[1,2]

Corresponding author: Daniel C Ducat
Email:ducatdan@msu.edu

This PDF file includes:

Figures S1 to S11
Legends for Movies S1 to S5
Legends for Tables S1 and S2

Other supporting materials for this manuscript include the following:

Movies S1 to S5
Tables S1 and S2

Figures

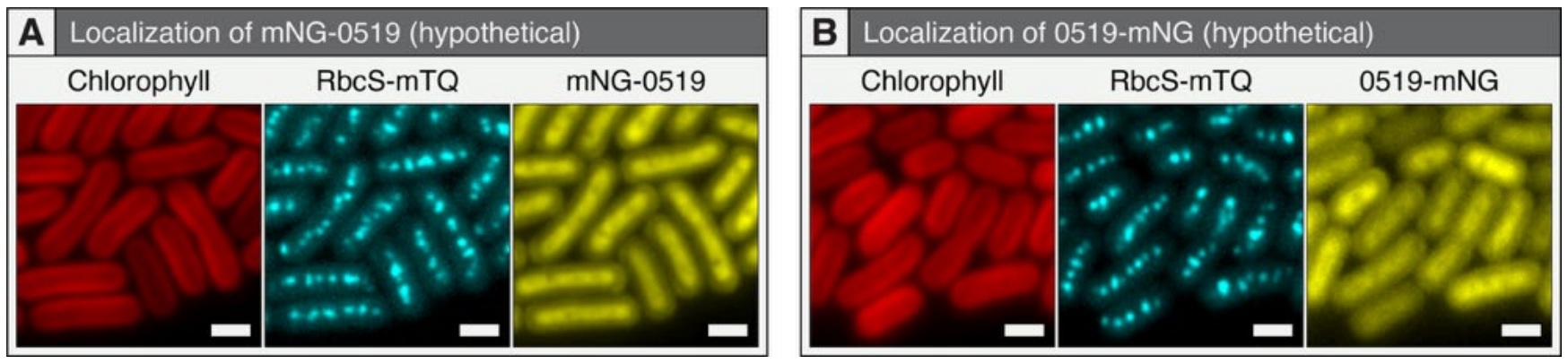

Figure S1. Synpcc7942_0519 does not exhibit co-localization with carboxysomes. (A) The endogenous genetic locus encoding Synpcc_0519 was modified to generate an N-terminal fusion with the fluorophore mNeonGreen (mNG-Synpcc7942_0519). The signal from this reporter appears diffuse through the cytosol with no evident co-localization with carboxysomes, as viewed by the Rubisco reporter, RbcS-mTQ. (B) A C-terminally tagged variant of this gene (Synpcc7942_0519-mNG) displayed a similar diffuse localization pattern. Scale bar = 2 μM.

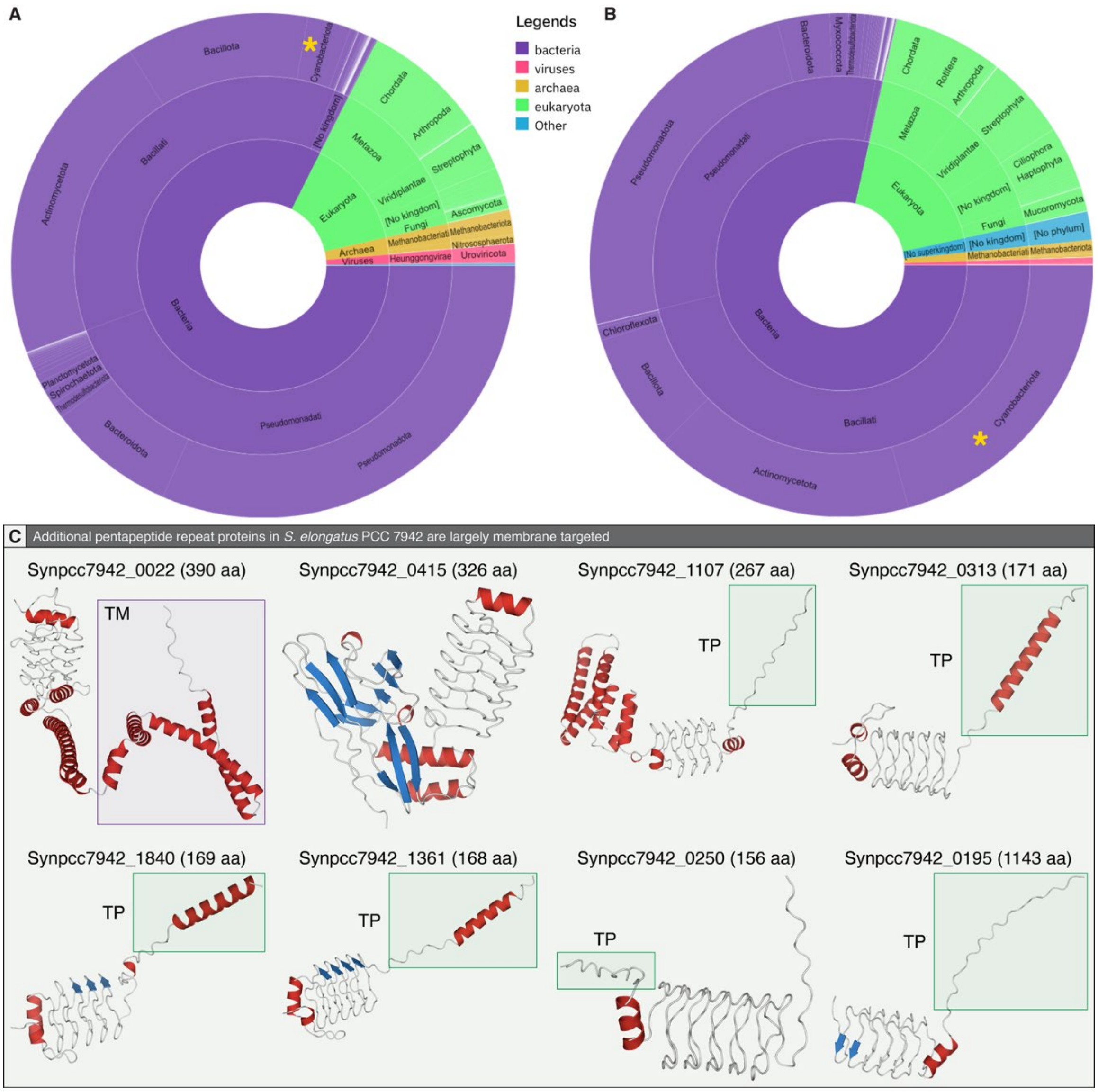

Figure S2. CipG belong to a larger family of pentapeptide repeat proteins. InterPro charts illustrating the (A) number of sequenced species and the (B) distribution of Pentapeptide Repeat (Pfam: PF00805) domain-containing sequences within the database. Sequences related to cyanobacterial genomes are starred with a yellow asterisk. (C) AlphaFold predicted structures for the eight additional pentapeptide repeat proteins found in *S. elongatus*. All predicted structures for the *S. elongatus* pentapeptide repeat proteins exhibit a right-handed quadrilateral β-helix. However, the length of the repeat domain varies. The N- and C-termini for these proteins are also varied and frequently do not have clear predicted structural features – which may be attributed to predicted transit peptide sequences (TP, green boxes) or transmembrane domains (TM, purple box) in some of these instances.

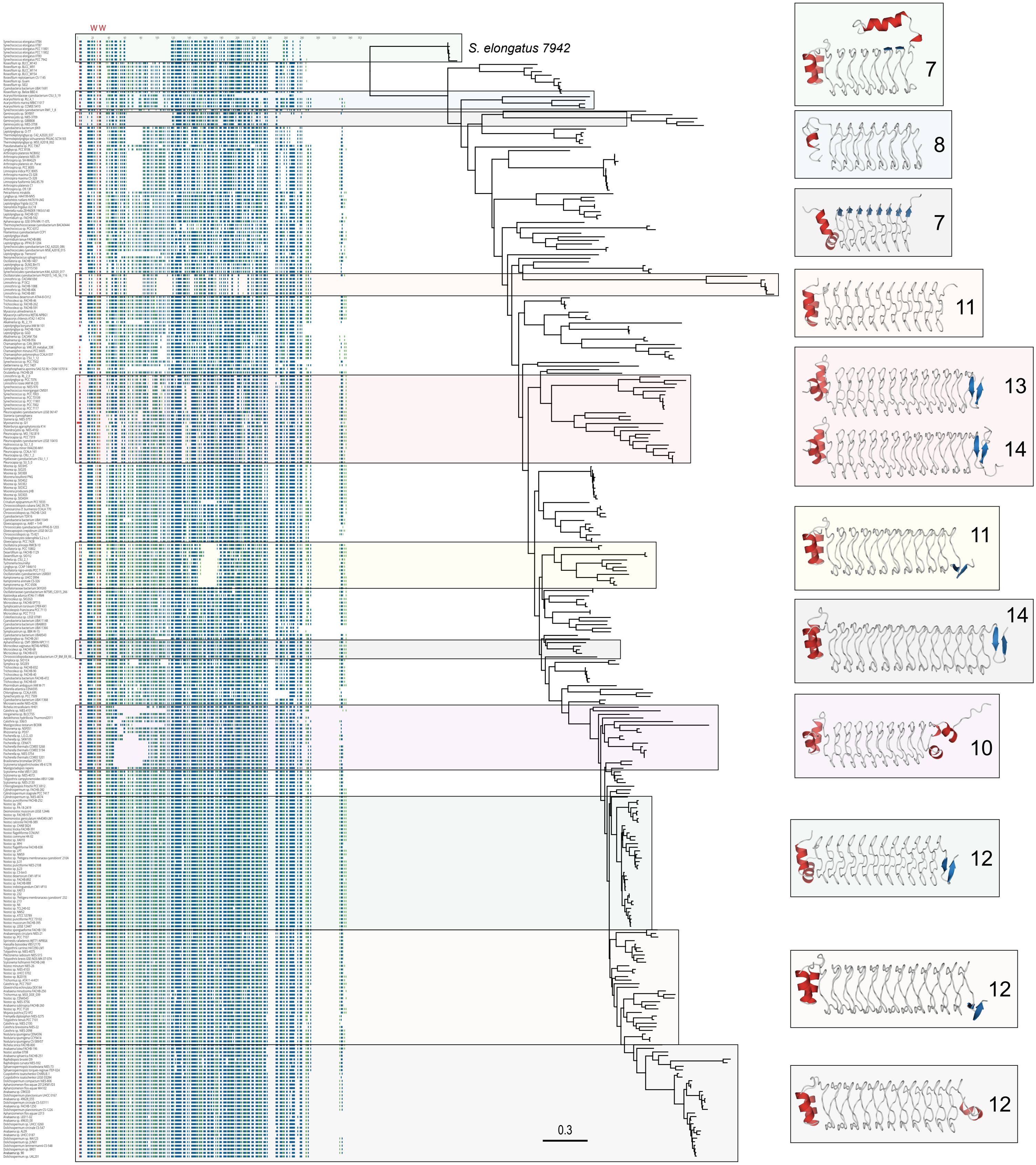

Figure S3. CipG alignments and inferred phylogeny highlight the distribution and quantity of AlphaFold predicted CipG coils. Alignment and phylogenetic tree representing relationship of >300 sequences of putative CipG homologs (co-localized in the genome with the locus encoding the carboxysome shell protein, CcmP). Note that the total number of tandem repeat domains varies across different species, contributing to predicted right-handed quadrilateral β-helix structures with between 7 and 14 full "turns." The S. elongatus sequence is marked in dark red. Note that because of the repetitive sequence features, the predicted phylogenetic relatedness between different CipG sequences is influenced strongly by the total number of tandem repeat domains.

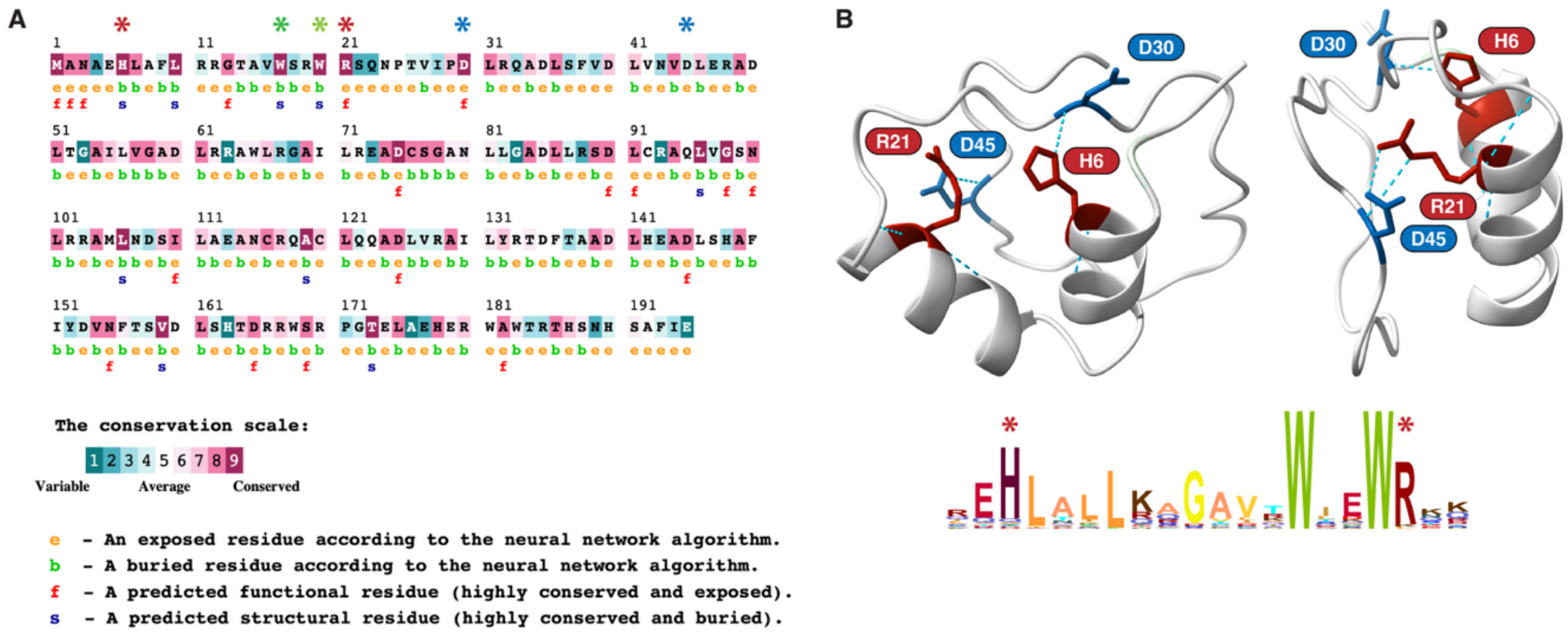

Figure S4. CipG N-terminal helices possess conserved residues. (A) *S. elongatus* CipG sequence with residues colored according to conservation across 445 CipG orthologs. The color of the asterisks denotes residues highlighted in panel B. (B) AlphaFold predicted structure and hydrogen bonding prediction for residues with CipG N-terminal helices.

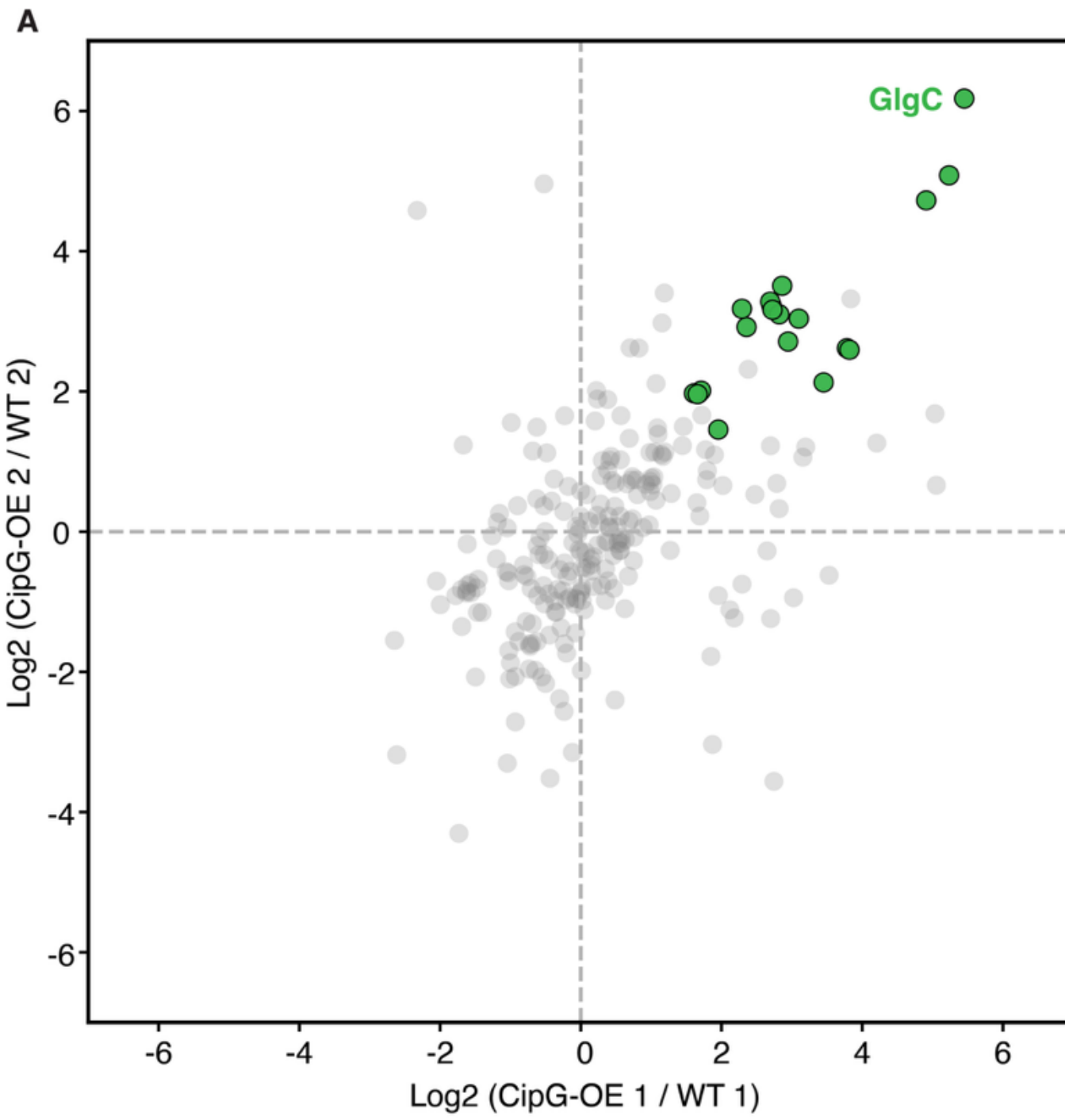

Gene	S1/C1	S2/C2	Q-value
GlgC	5.45	6.18	4.29e-02
NirA	5.23	5.08	4.29e-02
GatA	4.91	4.73	5.29e-02
RecA	2.86	3.51	1.20e-01
ChlP	2.71	3.23	1.20e-01
KaiC	2.69	3.28	1.20e-01
PGM1	2.95	2.71	1.21e-01
OGDH	3.78	2.62	1.21e-01
RpoC1	2.36	2.92	1.21e-01
SerA	3.82	2.59	1.45e-01
Gnd	2.82	3.10	1.55e-01
SpeE	2.29	3.18	1.58e-01
PurA	3.45	2.13	1.83e-01
CbbA	1.67	1.97	1.83e-01
SphX	2.72	3.16	1.83e-01
ClpR	1.71	2.01	1.83e-01
AtpG	1.61	1.97	1.83e-01
RpoB	1.66	1.96	1.83e-01
PilB	3.10	3.04	1.99e-01
IlvC	1.95	1.46	1.99e-01

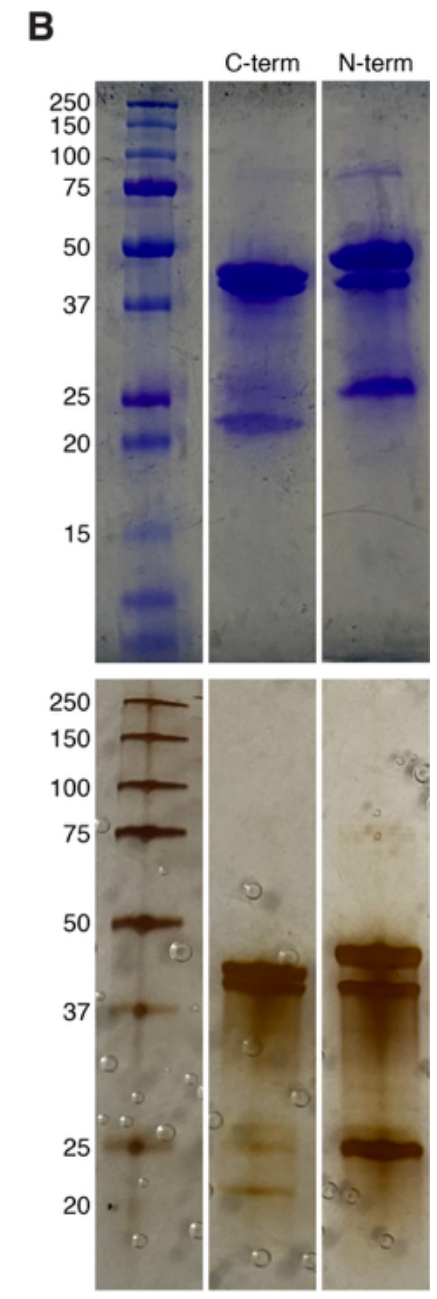

Figure S5. Co-immunoprecipitation of CipG-mNG reveals potential protein interaction partners. (A) Scatter plot comparing protein abundance in CipG–mNG Co-IP samples versus WT lysates across paired biological replicates. Each point represents a single protein plotted as $\log_2$-transformed abundance values for the corresponding sample–control pairs. The accompanying table lists the top-ranked enriched proteins, showing pairwise abundance ratios (S1/C1 and S2/C2) and the Benjamini–Hochberg FDR-corrected q value from a limma-style moderated statistical test. (B) Corresponding SDS-PAGE used for downstream band analysis of mNG-CipG and CipG-mNG fractions.

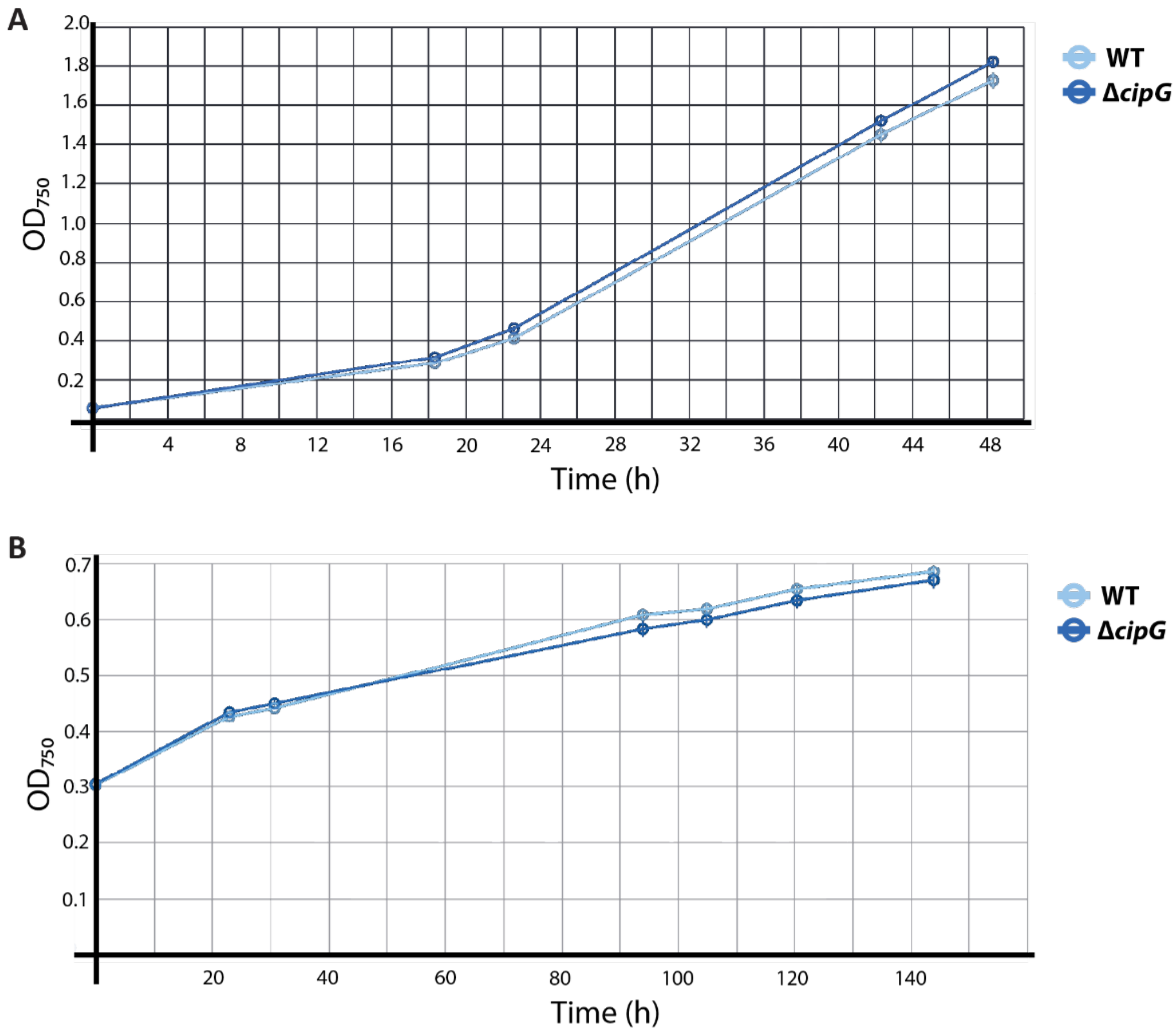

Figure S6. Deletion of *cipG* does not impair the ccm functions of the carboxysome. Representative growth curves of WT and Δ*cipG* strains under (A) high CO_2 and (B) ambient headspaces. Cultivation was performed in a Infors MiniTron incubator with a customized LED light array with illumination at ~50 µmol photons m^{-2} sec^{-1} and a 5600K light spectrum.

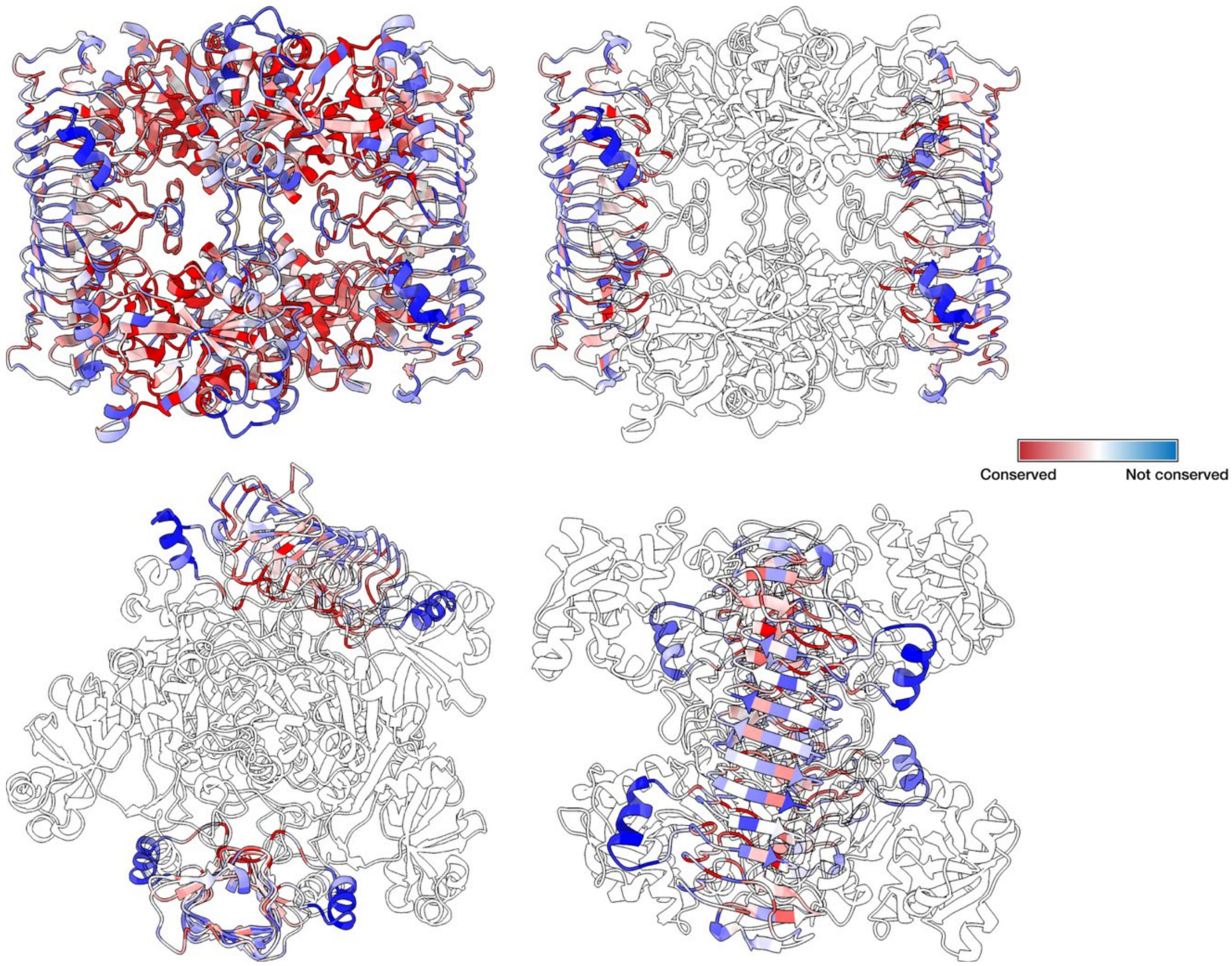

Figure S7. Structural features of cyanobacterial GlgC. (A) AlphaFold predicted structural views of GlgC from multiple angles, colored by residue conservation analysis across 608 cyanobacterial GlgC homologs.

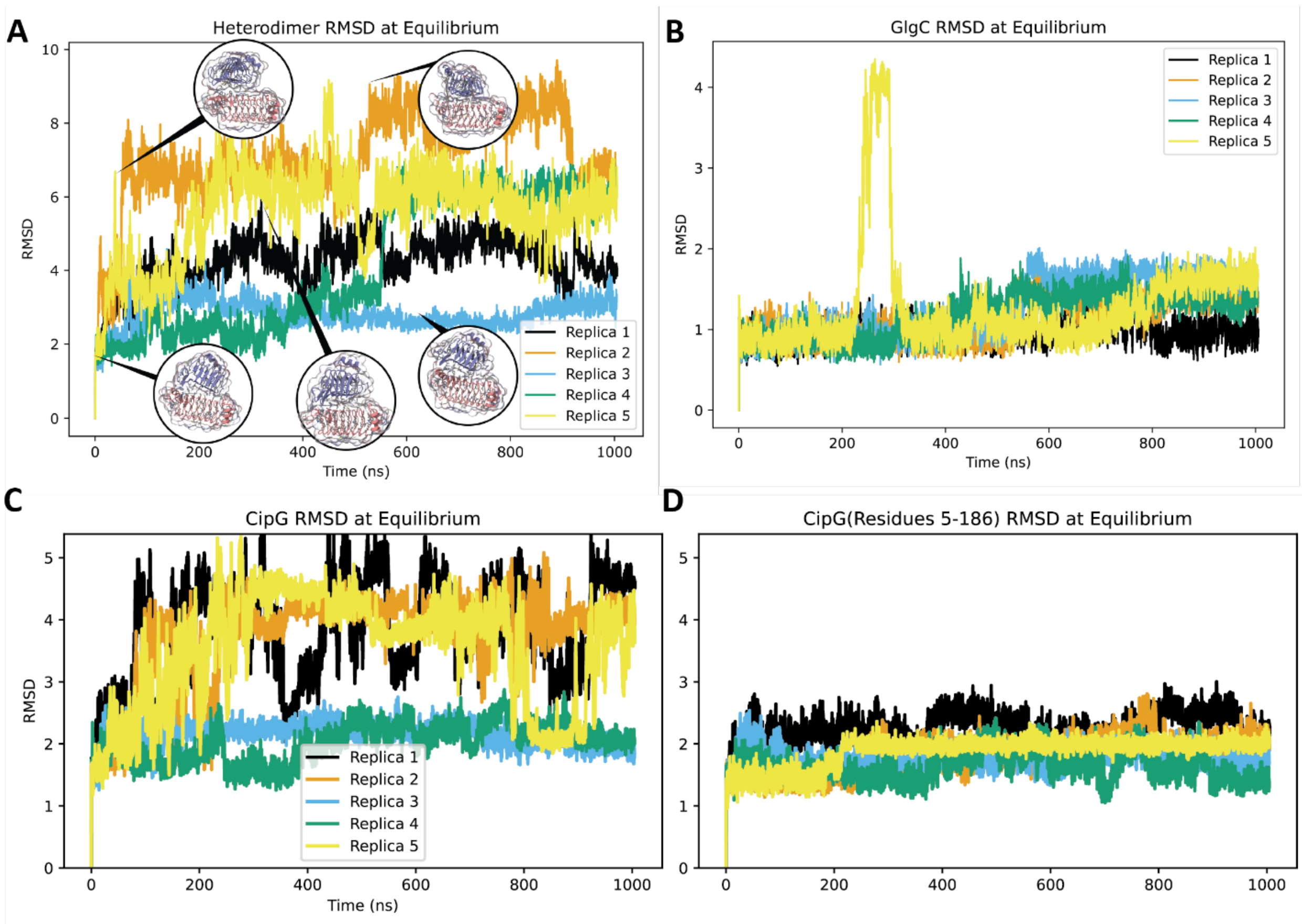

Figure S8. RMSD values of CipG and GlgC. (A) Calculated RMSD values for 5 replicates of a CipG-TPR dimer over the course of 1 µsec of simulation time. Representative conformational states of the CipG-TPR interface are shown as insets at the indicated time point for each simulation. (B) RMSD values calculated for the TPR domain of the GlgC monomer alone. (C) RMSD values calculated for the CipG monomer alone. A significant portion of the RMSD for CipG can be attributed to unstructured peripheral regions of CipG, as illustrated by (D) calculation of RMSD values for the core residues of CipG (*i.e.*, amino acids 5-186). The conformational shifts in amino acids outside of these core residues have no detectable influence on the CipG-TPR dimer interface.

A

CipG residues	Mean contacts per frame
Y132	7.2
Y152	6.1
W181	5.4
F150	4.6
R72	3.5
R185	3.3
R88	2.8
H178	2.7
R133	1.6
D153	1.6
Top 10	38.7
**All**	**56.1**

B

GlgC residues	Mean contacts per frame
R412	7.0
D382	6.2
S413	5.8
M346	4.5
K420	3.1
E401	2.7
E314	2.3
I311	2.3
I380	2.3
K383	2.1
Top 10	38.4
**All**	**56.1**

Figure S9. Predicted amino acid contacts contributing to the CipG-TPR interface. The ten most highly abundant contact sites between (A) CipG and (B) GlgC's TPR domain based on molecular dynamics simulations of the protein dimer.

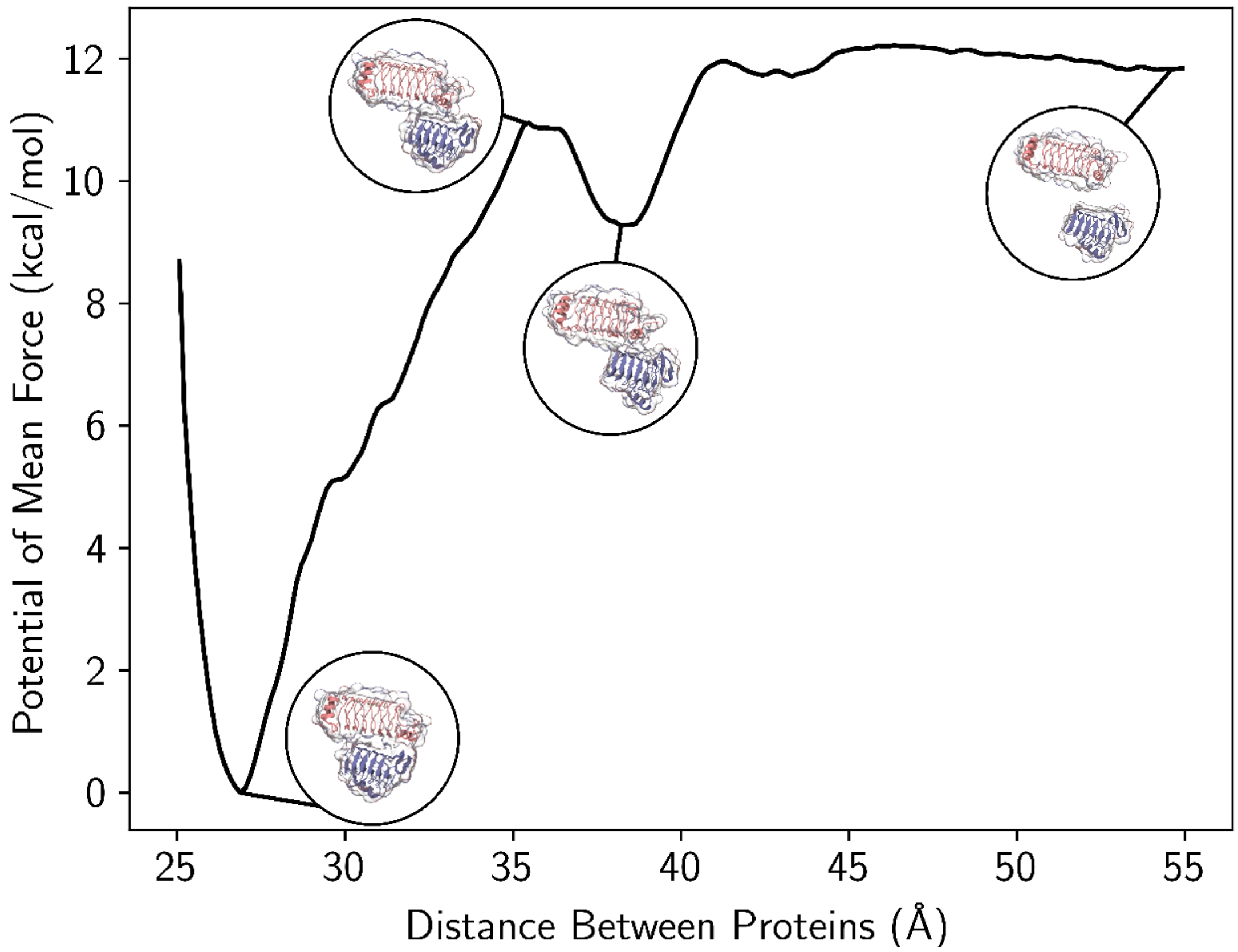

Figure S10. Energy landscape for steered simulations while separating CipG and the TPR domain of GlgC. Repeated sampling of the simulated transition states reveals a ~12.8 kcal/mol free energy change from the "bound state" (free energy trough near 27 Å distance) to the unbound proteins (>45 Å distance). Representative images of the protein interface between CipG and TPR domains are indicated with graphical insets.

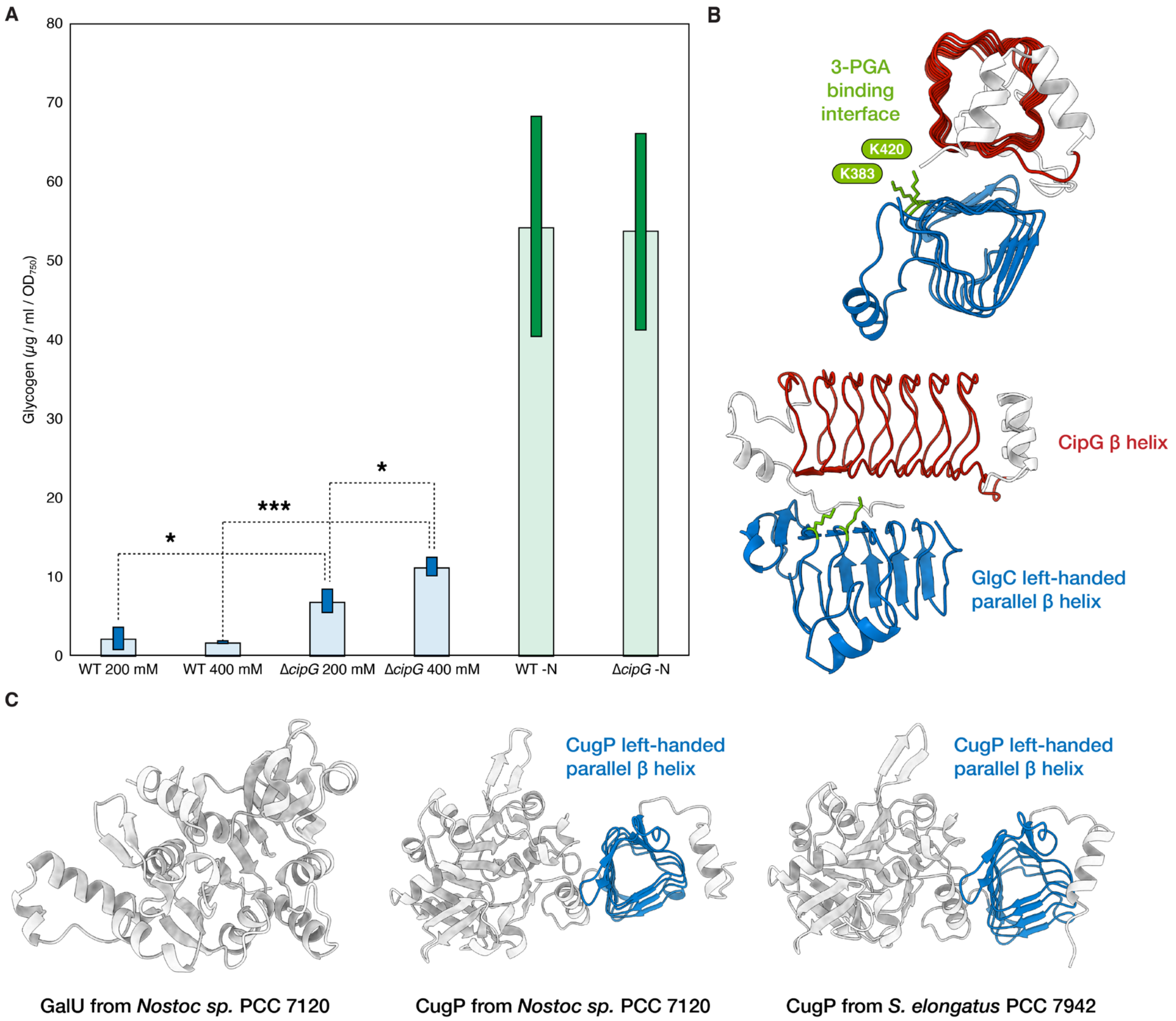

Figure S11. Additional sugar-utilizing enzymes possess GlgC-like β-helices. (A) Glycogen content is similarly elevated in both WT and Δ*cipG* mutants when grown in nitrogen deficient medium ($BG11_0$) (green bars). But glycogen content is significantly increased relative to WT in Δ*cipG* mutants under salt stress (200 mM and 400 mM NaCl, blue bars). Averages of 3 independent biological replicates are shown with ± SD reported with error bars. Significance was calculated by unpaired Student's t test. *$P < 0.05$, ***$P < 0.001$. (B) AlphaFold predicted binding between CipG (red) and the inward-facing left-banded β-helix of GlgC (blue). Residues critical for 3-PGA-binding highlighted in green. (C) AlphaFold Structural predictions for *Nostoc sp.* PCC 7120 GalU (left), CugP from *Nostoc sp.* PCC 7120 (middle), and CugP (right) from *S. elongatus* PCC 7942 (right).

Movie S1. 3D projection of Fig. 2A showing conservation of CipG residues.

Movie S2. 3D projection of Fig. S4B showing predicted hydrogen bonding residues of CipG N-terminal helices.

Movie S3. 3D projection of GlgC with regulatory β-helices highlighted in blue.

Movie S4. 3D projection of Fig. S7 showing residue conservation across 608 cyanobacterial GlgC homologs.

Movie S5. 3D projection of Fig. S7 highlighting residue conservation of β-helices across 608 cyanobacterial GlgC homologs.

Table S1. Protein sequences and species origins of the 445 putative homologs of CipG analyzed for this work.

Table S2. Raw proteomics dataset for proteins co-immunoprecipitated in pulldown experiments targeting CipG-mNG as bait.
